# Boosting thermo-photocatalytic CO_2_ conversion activity by using photosynthesis-inspired electron-proton-transfer mediators

**DOI:** 10.1038/s41467-020-20444-1

**Published:** 2021-01-05

**Authors:** Yingxuan Li, Danping Hui, Yuqing Sun, Ying Wang, Zhijian Wu, Chuanyi Wang, Jincai Zhao

**Affiliations:** 1 School of Environmental Science and Engineering, Shaanxi University of Science and Technology, Xi’an, 710021 China; 2 State Key Laboratory of Rare Earth Resource Utilization, Changchun Institute of Applied Chemistry, Chinese Academy of Sciences, Changchun, 130022 China; 3 Key Laboratory of Photochemistry, CAS Research/Education Center for Excellence in Molecular Sciences, Institute of Chemistry, Chinese Academy of Sciences, Beijing, 100190 China

**Keywords:** Photocatalysis, Electrocatalysis, Electrocatalysis

## Abstract

Natural photosynthesis proceeded by sequential water splitting and CO_2_ reduction reactions is an efficient strategy for CO_2_ conversion. Here, mimicking photosynthesis to boost CO_2_-to-CO conversion is achieved by using plasmonic Bi as an electron-proton-transfer mediator. Electroreduction of H_2_O with a Bi electrode simultaneously produces O_2_ and hydrogen-stored Bi (Bi-H_*x*_). The obtained Bi-H_*x*_ is subsequently used to generate electron-proton pairs under light irradiation to reduce CO_2_ to CO; meanwhile, Bi-H_*x*_ recovers to Bi, completing the catalytic cycle. This two-step strategy avoids O_2_ separation and enables a CO production efficiency of 283.8 μmol g^−1^ h^−1^ without sacrificial reagents and cocatalysts, which is 9 times that on pristine Bi in H_2_ gas. Theoretical/experimental studies confirm that such excellent activity is attributed to the formed Bi-H_*x*_ intermediate that improves charge separation and reduces reaction barriers in CO_2_ reduction.

## Abstract (web summary)

Using a single catalyst to mimic the two-step photosynthesis for CO_2_ conversion remains a challenge. Here, the authors report a simple Bi catalyst that can act as an electron-proton-transfer mediator to spatially and temporally separate H2_O_ splitting and CO_2_ reduction reactions in CO_2_-to-CO conversion process.

## Introduction

Catalytic CO_2_ reduction driven by solar light or renewable electricity to produce useful fuels is a promising strategy for solving energy and greenhouse effect issues^1^. The CO_2_ reduction activity is usually limited by the rate-limiting step of transferring an electron to a linear CO_2_ molecule to form a bent CO_2_^•−^ anion radical (Eq. 1)^1–3^. After the rate-determining step, the intermediate CO_2_^•−^ is subsequently reduced via the proton-assisted electron transfer approach, in which H_2_ or H_2_O is utilized as the proton source^1–3^. In these CO_2_ reduction processes, the types of the products are determined by the number of the transferred proton–electron pairs (Eqs. 2–5)^2^:

1 $${\mathrm{CO}}_2 + {\mathrm{e}}^ - = {\mathrm{CO}}_2^{ \bullet - },$$

2 $${\mathrm{CO}}_2 + 2{\mathrm{H}}^ + + 2{\mathrm{e}}^ - = {\mathrm{CO}} + {\mathrm{H}}_2{\mathrm{O}},$$

3 $${\mathrm{CO}}_2 + 4{\mathrm{H}}^ + + 4{\mathrm{e}}^ - = {\mathrm{HCHO}} + {\mathrm{H}}_2{\mathrm{O}},$$

4 $${\mathrm{CO}}_2 + 6{\mathrm{H}}^ + + 6{\mathrm{e}}^ - = {\mathrm{CH}}_3{\mathrm{OH}} + {\mathrm{H}}_2{\mathrm{O}},$$

5 $${\mathrm{CO}}_2 + 8{\mathrm{H}}^ + + 8{\mathrm{e}}^ - = {\mathrm{CH}}_4 + 2{\mathrm{H}}_2{\mathrm{O}}.$$

Although inexpensive and abundant H_2_O is generally believed to be the ideal proton source^4^, light-driven CO_2_ reduction with pure water is hampered by the difficulties in accumulating photogenerated charges and appropriately coupling two half-reactions on a single catalyst^5,6^. Furthermore, the present photocatalytic CO_2_ reductions with H_2_O are universally carried out in a single reactor^7,8^, and the obtained gases are mixtures of hydrocarbons and O_2_. The presence of O_2_ inevitably leads to oxygen contamination and may also improve the oxidation of the hydrocarbons that significantly reduce the efficiency. As a result, the practical separation of O_2_ from the mixed gas becomes a serious technological and economic issue for large-scale and sustainable applications of CO_2_ conversion^9^. To overcome the above problems, photocatalytic CO_2_ reductions are usually carried out by using H_2_ as a proton source^10^. Under light irradiation, the adsorbed H_2_ molecules are first dissociated into hydrogen adatoms (H_2_* → 2H*, where * represents the active site), which immediately react with the photogenerated holes to form H^+^ on the catalyst surface^11^. Then, CO_2_ reduction can be facilitated by a proton-coupled electron transfer process with a much lower potential^3^. Compared with H_2_O, the relatively high cost and explosive feature of H_2_ may be two drawbacks for its application in CO_2_ conversion. Furthermore, the H_2_ dissociation process inevitably requires an energy input, whose value depends on the used catalysts. Based on the above discussion, we can conclude that both H_2_ and H_2_O have their own unique sets of advantages and shortcomings in CO_2_ reduction. A CO_2_ conversion strategy that can simultaneously avoid the drawbacks of H_2_ and H_2_O might be revolutionary in catalytic transformation. Above all, significant opportunities for redesigning the catalytic strategy to convert CO_2_ for practical applications are believed to exist.

**Fig. 1:**
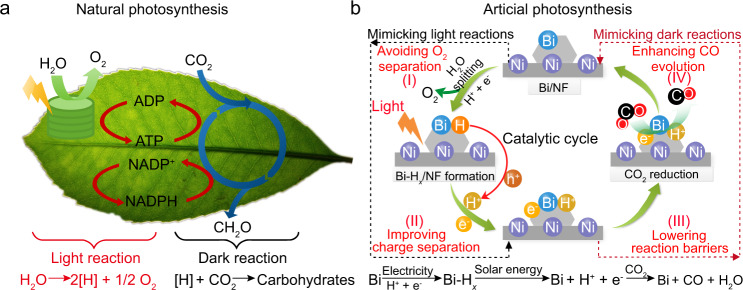
Photosynthesis in nature and the artificial analogy in this study. **a** Schematic depiction of the light and dark reactions in natural photosynthesis. **b** Graphical representation of the reaction pathway of the artificial CO_2_ reduction with the benefits of the reaction model.

Unlike traditional artificial CO_2_ reduction by a one-step reaction, natural photosynthesis in green plants can convert CO_2_ and H_2_O into carbohydrates through two sequential steps, which are known as the light and dark reactions (Fig. 1a). Under sunlight irradiation, chloroplasts can enable the synthesis of reducing equivalents (i.e., [H]) and O_2_ from water splitting^12,13^. Then, the reducing equivalents are used to produce reduced nicotinamide adenine dinucleotide phosphate (NADPH) and adenosine triphosphate (ATP) by sequential e^−^ and H^+^ transfer steps, respectively. In the dark reaction, with the help of NADPH, H^+^, and ATP, CO_2_ reduction can be carried out stepwise to generate carbohydrates^12^. As a result, the water-splitting process is temporally and spatially separated from CO_2_ reduction reactions by forming a reductive intermediate, which is helpful in promoting CO_2_ conversion by lowering the reaction barrier and avoiding charge accumulation and O_2_ separation^14,15^. Therefore, natural photosynthesis provides a two-step reaction model for innovative catalyst design in CO_2_ conversion with H_2_O.

Similar to the natural photosynthesis, the decoupled approach for water splitting has been explored recently, in which water oxidation and proton reduction reactions were spatially separated^16,17^. Compared with water splitting, CO_2_ reduction is much more difficult because CO_2_ is a thermodynamically stable molecule with a linear structure^1^. Furthermore, finding a single material to mimic natural photosynthesis for CO_2_ conversion is more challenging because it should work as a redox shuttle to bridge the separated water splitting and CO_2_ reduction reactions^12,18^, which is more complicated than the only water splitting process. Here, we present a design strategy to realize these sequential reactions using a single Bi catalyst (Fig. 1b). First, splitting of H_2_O into H atoms and O_2_ and storage of the H atoms in Bi nanoparticles (denoted as Bi-H_*x*_) were simultaneously achieved by an electrochemical approach. Then, the obtained Bi-H_*x*_ was used as a reducing equivalent to reduce CO_2_ to CO by in situ generating H^+^/e^−^ pairs under light irradiation. Meanwhile, Bi-H_*x*_ recovered to Bi for reversible storage and release of hydrogen. In this process, Bi can function as an electron–proton-transfer mediator to bridge water splitting and CO_2_ reduction reactions. A detailed mechanism for CO production with the benefits of this strategy is shown in Fig. 1b. This reaction model provides a concept for developing efficient and multifunctional catalysts for CO_2_ conversion by mimicking photosynthesis.

## Results

**Fig. 2:**
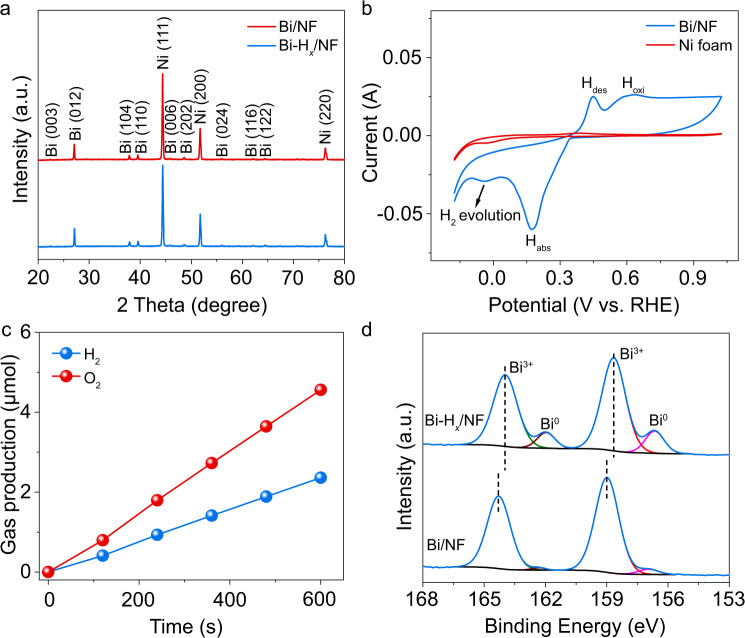
Preparation and characterization of Bi with and without stored hydrogen. **a**, **d** XRD patterns, and high-resolution Bi 4*f* XPS spectra of Bi/NF and Bi-H_*x*_/NF. **b** Cyclic voltammograms of the as-prepared Bi/NF and pristine NF in 1 M KOH solution. Scan rate: 50 mV s^−1^. **c** H_2_ and O_2_ evolution curves on the Bi/NF electrode by water splitting at −0.18 V (vs. RHE).

Bi was loaded on nickel foam (NF) by an electrodeposition method. The NF was chosen as a substrate for depositing Bi because of its good mechanical performance and high porosity with an interconnected framework structure that is favorable for easy contact between electrolyte and electrode, and fast ion transport. As shown in Fig. 2a (red line), the diffraction peaks for Bi/NF can be assigned to the rhombohedral Bi structure (JCPDS#44-1246) and NF. The quality of the loaded Bi was determined to be 1.253 mg by inductively coupled plasma (ICP) atomic emission spectroscopy. Cyclic voltammetry (CV) curves of the porous nickel and Bi/NF electrodes in 1 M KOH electrolyte are shown in Fig. 2b. In this work, all the potentials were given by the reversible hydrogen electrode (RHE). For the NF electrode (red line in Fig. 2b), the peaks at ca. −0.03 and 0.38 V vs. RHE (vs. RHE) can be attributed to adsorption and desorption of hydrogen, respectively^19^. When Bi/NF was used as the working electrode (blue line in Fig. 2b), two cathodic adsorption/reduction and H_2_ evolution peaks of hydrogen are obviously observed at ca. 0.17 (indicated by H_ads_) and −0.04 V, respectively. During the following anodic polarization of Bi/NF, two current peaks can be observed at ca. 0.45 and 0.62 V (blue line in Fig. 2b), which are ascribed to hydrogen desorption (H_des_) and hydrogen oxidation (H_oxi_) on the Bi electrode, respectively^20^. Hydrogen desorption occurs before hydrogen oxidation, suggesting strong adsorption of hydrogen on the surface of Bi/NF^19^. Such behavior is not observed in the CV curve of NF in Fig. 2b, indicating that the presence of Bi is responsible for the electrochemical hydrogen storage behavior of Bi/NF. Similar hydrogen storage properties of Bi in acid solution have also been studied previously^21^.

Electrochemical hydrogen storage in Bi was performed at a reduction potential of −0.18 V for 10 min, and the obtained sample is denoted Bi-H_*x*_. In this process, the H_2_ and O_2_ evolutions by water splitting are shown in Fig. 2c. The obtained products exhibit a nearly linear rise in time from 0 to 10 min. It is worth noting that the molar ratio of H_2_/O_2_ is ~0.52, which is much lower than the stoichiometric ratio of 2. This result suggests that a part of the reduced H species was stored in Bi electrode without desorption, which is consistent with the CV curve of Bi/NF in Fig. 2b. The number of hydrogen atoms (M_H_) stored in Bi-H_*x*_/NF was estimated to be 13.52 μmol based on the amounts of the produced H_2_ (${\mathrm{M}}_{{\mathrm{H}}_{2}}$) and O_2_ (${\mathrm{M}}_{{\mathrm{O}}_{2}}$) in Fig. 2c (M_H_ = 4${\mathrm{M}}_{{\mathrm{O}}_{2}}$  − 2${\mathrm{M}}_{{\mathrm{H}}_{2}}$). Moreover, Faradaic efficiency (FE) of the Bi electrocatalyst for H_2_O splitting was calculated to be 20.9% based on the O_2_ evolution according to Eq. (7) in the “Methods” section.

The X-ray diffraction (XRD) pattern of the Bi-H_*x*_ product is shown in Fig. 2a (blue line), which is similar to that of pure Bi. This result indicates that the crystal structure of Bi was not affected by hydrogen storage. However, hydrogen storage in a metal may affect the electronic structure of the metal via electron donation. Therefore, X-ray photoelectron spectroscopy (XPS) studies were performed to study the electronic structures of Bi/NF and Bi-H_*x*_/NF. The high-resolution spectra of Bi 4*f* are presented in Fig. 2d. As shown in Fig. 2d, the XPS spectra of the two samples can be deconvoluted into four peaks related to metallic Bi and the oxidation state of Bi. The oxidation state of Bi was formed by surface oxidation during the exposure of Bi to air^22,23^. For Bi/NF, the binding energies at 159.0 and 164.2 eV are assigned to the Bi^3+^ of pure Bi_2_O_3_^23^. However, for Bi-H_*x*_/NF, the peaks belonging to the oxidation states of Bi are obviously red-shifted relative to those for Bi/NF, which is related to incomplete oxidation of Bi due to the electron donation of hydrogen atoms. High-resolution XPS spectra of O 1*s* for the two samples were examined to further confirm the surface chemistry of Bi (Supplementary Fig. 1). The two samples exhibit similar O 1*s* XPS spectra that can be deconvoluted into three peaks corresponding to, Bi-O bands (529.3 eV), surface hydroxyl oxygen (530.8 eV), and adsorbed O_2_ (532.7 eV), further confirming the generation of Bi-O in Bi_2_O_3_ on the surface of the two samples^24^. The results of the XPS study are consistent with the fact that hydrogen storage in Bi occurs during the electrochemical treatment process. Notably, the main body of Bi/NF or Bi-H_*x*_/NF is still composed of metallic Bi based on the XRD patterns in Fig. 2a.

**Fig. 3:**
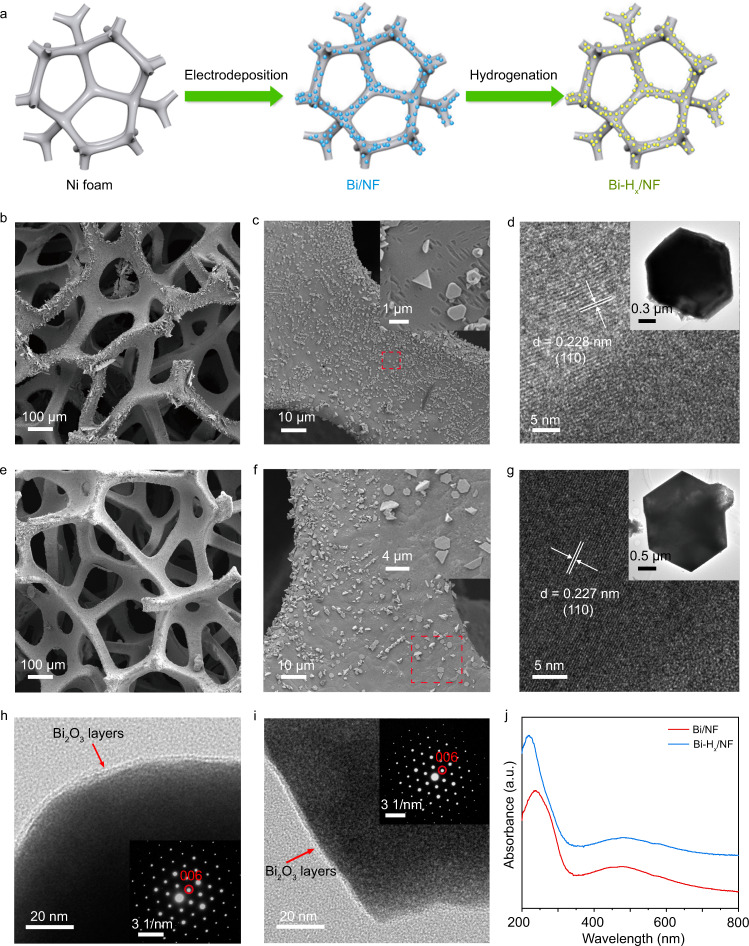
Morphologies, microstructures, and light absorption properties of the samples. **a** Schematic illustration of the synthesis process of Bi-H_*x*_/NF. SEM images of (**b**, **c**) Bi/NF and (**e**, **f**) Bi-H_*x*_/NF. The insets in (**c**) and (**f**) are enlarged views of the corresponding SEM images marked in red squares. **d**, **g** HRTEM images of Bi/NF and Bi-H_*x*_/NF. The insets in (**d**) and (**g**) are the TEM images of Bi/NF and Bi-H_*x*_/NF sheets, respectively. **h**, **i** TEM images of the edges of Bi/NF and Bi-H_*x*_/NF sheets for showing the formation of amorphous layers. The insets in (**h**) and (**i**) are the SEAD patterns of Bi/NF and Bi-H_*x*_/NF. **j** UV–visible absorption spectra of Bi/NF and Bi-H_*x*_/NF.

Based on the results in Fig. 2, the loading process of Bi-H_*x*_ on NF is schematically shown in Fig. 3a. The morphology of the as-prepared samples was visualized by scanning electron microscopy (SEM). The SEM images of Bi/NF and Bi-H_*x*_/NF in Fig. 3b, e show that the NF has a macroporous structure with pore sizes between 100 and 400 μm. The magnified SEM images in Fig. 3c, f indicate that both NF skeletons are covered with hexagonal sheets with a size of 0.5–2 μm, proving that Bi was successfully deposited on the surface of NF and that the hydrogen storage process did not change the morphology of Bi. The microstructures of the nanosheets were further characterized by transmission electron microscopy (TEM) and selected area electron diffraction (h). As shown in Fig. 3d, g, high-resolution TEM (HRTEM) images of the hexagonal nanosheets display crystal lattices corresponding to the (110) plane of primary Bi. As shown by the XPS spectra in Fig. 2d, the surface of Bi can be easily oxidized into Bi_2_O_3_ in the air. To investigate the formed Bi_2_O_3_ layer, the TEM analysis was performed on the edge of the Bi-H_*x*_ and Bi sheets. As shown in Fig. 3h, i, amorphous Bi_2_O_3_ layers with a thickness of ~3 nm were clearly formed on both surfaces of Bi-H_*x*_ and Bi. The SAED patterns of Bi (inset of Fig. 3h) and Bi-H_*x*_ (inset of Fig. 3i) show that only the spots ascribing to the rhombohedral Bi are observed, suggesting the single-crystalline nature of the samples. No distinct change in the microstructure is found for the sample after hydrogen storage.

Then, ultraviolet (UV)–visible absorption spectroscopy was used to study the optical properties of the samples (Fig. 3j), with the results showing that hydrogen storage does not obviously change the light absorption of Bi. As shown in Fig. 3j, the light absorption spectra of the two samples reach ~600 nm, proving that Bi/NF is a good candidate for absorbing the visible light of solar radiation. In addition, both samples display an absorption peak in the range between 400 and 600 nm. To prove that this peak is caused by the localized surface plasmon resonance (LSPR) absorptions, the Bi nanostructures with sizes of ~100 and ~400 nm were further synthesized at the deposition time of 1 and 4 min, respectively (Supplementary Fig. 2). XRD measurements show that elemental Bi was formed in these situations (Supplementary Fig. 3a). Then, the corresponding absorption spectra of the obtained Bi/NF at different deposition times were tested (Supplementary Fig. 3b). The Bi sample with a diameter of ~100 nm shows a bandgap absorption onset of ~600 nm, whereas the absorption peak at ~480 nm in Fig. 3h disappears. This optical phenomenon implies that the absorption peak between 400 and 600 nm in Fig. 3h was induced by LSPR, rather than from the coupling effect of Bi and Bi_2_O_3_ layer. Moreover, by comparing the spectra of Bi samples prepared at 4 and 10 min, we can find that the position of the absorption peak shows a red-shift with the size increasing (Supplementary Fig. 3b), which is consistent with the characteristic of LSPR absorption that is strongly correlated to the shape and size of the materials^25^. The above optical properties of the samples confirm that the absorption peak at 480 nm in Fig. 3h can be attributed to the LSPR of Bi, which is consistent with the previous report^26^.

Coupling of plasmonic Bi nanoparticles can extend the light absorption of semiconductor photocatalysts and achieve enhanced activity^27^. However, direct photocatalysis on plasmonic Bi nanostructures, which is termed as plasmonic photocatalysis, has rarely been reported. Compared with the semiconductor-based photocatalysis that has received the most attention for several decades, plasmonic photocatalysis just started from the year 2011^28^. Therefore, plasmonic metallic nanostructures represent a new family of photocatalysts, and even a few reports focus on CO_2_ reduction^29^. In plasmonic photocatalysis, a relatively high reaction temperature (>150 °C) is generally required for activating the adsorbed molecules^29–31^. Therefore, it is reasonable that the photocatalytic CO_2_ reduction on the plasmonic Bi should be triggered by heat input. Although most plasmonic metals used as a light absorber in metal-semiconductor systems can induce photocatalytic reactions at room temperature, the low charge transfer (CT) efficiency between metals and semiconductors is the key to limit their applications^32^. Furthermore, most of the plasmonic photocatalysts for CO_2_ reductions are based on noble metal nanoparticles, such as Ag, Au, and Rh^30,31^.

**Fig. 4:**
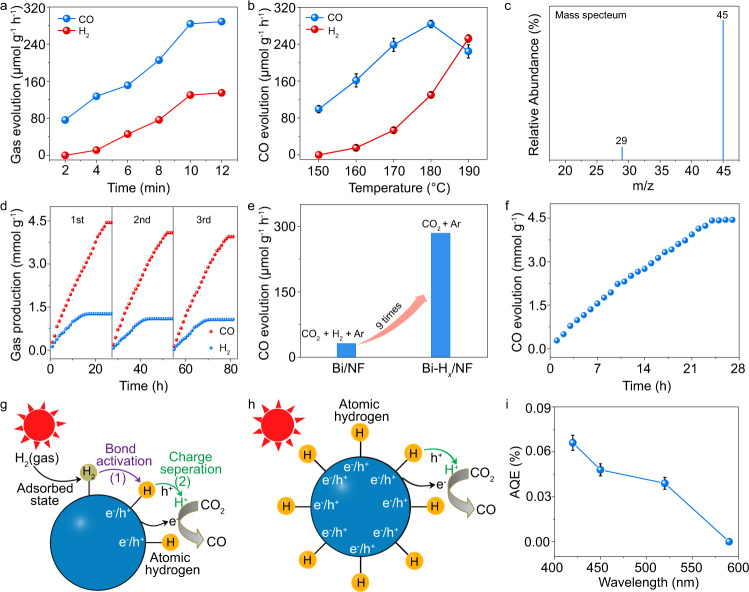
Thermo-photocatalytic CO evolution tests. **a** Reduction of CO_2_ over Bi-H_*x*_/NF catalysts treated in KOH for different times. **b** Effect of temperature on CO and H_2_ evolution rates over Bi-H_*x*_/NF under a 420-nm LED light irradiation (*n* = 3, error bars: standard deviation). **c** Mass spectrum from GC-MS analysis of the CO generated in the catalytic CO_2_ reduction reaction using ^13^CO_2_. **d** Stability test of Bi-H_*x*_/NF using three repeats of the CO_2_ reduction reaction under 420-nm LED light irradiation at 180 °C. **e** Comparison on the CO production activities between Bi/NF using H_2_ as proton source and Bi-H_*x*_/NF using the stored H as a proton source. **f** CO evolution curve on the Bi-H_*x*_/NF catalyst treated in the air at 80 °C for 0.5 h. **g**, **h** Schematic diagram of CO_2_ reduction processes using H_2_ as proton source on Bi/NF and preformed H species as proton source on Bi-H_*x*_/NF. **i** AQEs of CO evolution over the Bi-H_*x*_/NF catalyst under different wavelength irradiation (*n* = 3, error bars: standard deviation).

After the electrochemical hydrogen storage process of Bi/NF in 1 M KOH electrolyte, the formed Bi-H_*x*_/NF was used for CO_2_ reduction in another gas–solid reactor with a quartz window on top, in which both the temperature and light illumination could be controlled. Initially, the reduction of CO_2_ over Bi-H_*x*_/NF treated in KOH for 2–12 min was investigated at 180 °C by monitoring the production of CO (Fig. 4a). Other possible products (such as CH_4_ and CH_3_OH) were not detected, confirming that high selectivity for CO_2_ reduction was achieved on Bi-H_*x*_/NF. As shown in Fig. 4a, the catalyst treated for 10 min shows the highest CO formation rate, and similar activity for producing CO is exhibited when the reaction time is further increased to 12 min. Therefore, we focused our study on the sample treated for 10 min to investigate the roles of the stored hydrogen in the CO_2_ reduction process.

Figure 4b shows the CO and H_2_ evolution activity of Bi-H_*x*_/NF at different temperatures under 420 nm light-emitting diode (LED) light irradiation with low intensity (108.6 mW cm^−2^). As shown in Fig. 4b, 100% selectivity towards CO production (99.0 μmol g^−1^ h^−1^) is achieved on Bi-H_*x*_/NF at 150 °C. When the reaction temperature reaches 160 °C, H_2_ evolution is observed, which increases with increasing temperature. Enhancing the reaction temperature can induce an increase in the relative population of adsorbed CO_2_ molecules in excited vibrational states based on the Bose–Einstein distribution^33^. Due to this effect, a higher temperature is required to facilitate activation and subsequent reduction of CO_2_ molecules, as shown in Fig. 4b. Because no proton sources were added into the reactor, the H_2_ evolution behavior should be caused by the release of H_2_ from Bi-H_*x*_/NF upon heating, which is easy to understand according to the simple Le Chatelier’s principle. The desorption of H_2_ molecules from metals with stored hydrogen induced by the heating effect has been extensively studied experimentally^34^. As shown in Fig. 4b, the CO production rate increases with temperature and reaches a maximum of 283.8 μmol g^−1^ h^−1^ at 180 °C. Significantly, this high CO production rate on the simple Bi catalyst is achieved without the use of any cocatalysts. Therefore, we can conclude that the two-step strategy holds great potential in CO_2_ reduction with H_2_O. As shown in Fig. 4b, a further increase in temperature reduces the CO evolution rate because many more reactive H atoms are converted to H_2_ molecules. The H_2_ release event further demonstrates that H atoms were indeed stored in the Bi nanocrystals, resulting in the formation of a reductive surface on Bi that can cause CO_2_ reduction.

As a control experiment, pristine NF was treated at a reduction potential of −0.18 V for 10 min in 1 M KOH electrolyte and then placed in the gas–solid reactor for testing the thermo-photocatalytic CO_2_ reduction activity. Under identical reaction conditions to Bi-H_*x*_/NF, no CO production is detected after 4 h of reaction, indicating a critical role of Bi-H_*x*_ in driving photocatalytic CO_2_ reduction. Control experiments in the absence of light, heat, CO_2_, or catalyst were further performed, and the CO product is not detected, demonstrating that CO production on Bi-H_*x*_/NF is driven by CO_2_ reduction under the synergetic effect of photon flux and thermal energy.

Considering that the heat input can be provided by the infrared light of sunlight (accounting for ~50% of the solar energy), thus the plasmonic Bi photocatalysts can potentially use the entire solar spectrum for driving catalytic reactions by employing a high-temperature solar reactor driven by concentrated solar radiation. Although conventional semiconductor photocatalysts can work at room temperature, their activities always decreased with increased temperatures due to the relatively low Debye temperatures of the semiconductors^30,35^. Therefore, in practical applications, the heat effect from concentrated sunlight might be one of the hindrances for efficient solar energy conversion in gas–solid reaction system by using semiconductor photocatalysts^30^. In this respect, the ability of the plasmonic Bi photocatalyst that can work at 180 °C might have its own advantage for CO_2_ reduction.

Moreover, an isotopic ^13^CO_2_ labeling experiment was also carried out on Bi-H_*x*_/NF under identical reaction conditions (Fig. 4c) on the basis of gas chromatography (GC)-mass spectrometry. The peak attributed to the Ar gas was not provided in Fig. 4c because the high intensity of it disturbed the distinguishing of other low-intensity peaks. A peak is observed at *m*/*z* = 29 (^13^CO), further confirming that the generated CO originates from the light-induced reduction of CO_2_ rather than from contaminants^7^. For comparison, the isotopic ^12^CO_2_ labeling experiment was also performed on Bi-H_*x*_/NF (Supplementary Fig. 4), and ^12^CO with *m*/*z* = 28 is found to be the main product for ^12^CO_2_ reduction, which further demonstrates that CO was produced from the thermo-photocatalytic reduction of CO_2_ on Bi-H_*x*_/NF. As shown in Fig. 4c, except for the peaks belonging to CO_2_ and CO, no other peaks were observed in Fig. 4c, further confirming the high selectivity for CO_2_ reduction on Bi-H_*x*_/NF.

To prove the recycling capacity of Bi-H_*x*_/NF, repeated CO_2_ reduction experiments under 420-nm LED light irradiation were carried out (Fig. 4d). After each run, the Bi-H_*x*_/NF was rehydrogenated by the same electrochemical method (reacting in 1 M KOH solution at a voltage of −0.18 V for 10 min) and added into the reactor with CO_2_ gas. As shown in Fig. 4d, the CO evolution reaction stops after nearly 27 h of reaction in each run because the surface of Bi-H_*x*_/NF loses the ability to reduce CO_2_ with the consumption of the stored hydrogen atoms. In this process, Bi-H_*x*_ gradually recovers to Bi, which can be further used for H storage and CO production, as shown in the second and third runs in Fig. 4d. This observation indicates that the stored hydrogen indeed plays an important role in CO_2_ reduction. In the third run, the CO evolution on Bi-H_*x*_/NF can still maintain ~90% of the initial activity after 81 h of reaction, suggesting that Bi-H_*x*_/NF is stable and that CO_2_ reduction on Bi-H_*x*_ can be achieved by a catalytic cycle. The XRD pattern and an SEM image of Bi-H_*x*_/NF after 81 h of photocatalytic reaction were obtained (Supplementary Fig. 5), and no apparent changes in the crystal structure or morphology are observed, indicating that Bi-H_*x*_/NF has favorable chemical stability. These results confirm that Bi/NF can function as a reversible hydrogen storage material to complete the catalytic cycle.

It is well known that H_2_ is traditionally used as the proton source for photochemical CO_2_ conversion via the proton-assisted electron transfer approach^3^. In order to show that the current approach using surface-bound H atoms as a proton source has advantages over the traditional H_2_, the controlled experiment for CO_2_ reduction was carried out on Bi/NF material in dissociative H_2_ gas (0.5 mL). As shown in Fig. 4e, although the number of hydrogen atoms stored in the Bi-H_*x*_/NF systems (13.52 μmol) is only ~25% of that from H_2_ in Bi/NF reaction system, the CO_2_ reduction activity of Bi-H_*x*_/NF reaches nine times that of Bi/NF. This result indicates that Bi-H_*x*_/NF has great utility for achieving high activity in catalytic CO_2_ reduction by concentrating reactive hydrogen adatoms on the catalyst.

As shown in the XPS spectra in Fig. 2d, the amount of Bi^0^ species on the surface of Bi-H_*x*_/NF is larger than that of Bi/NF. In order to exclude that the enhanced activity of Bi-H_*x*_/NF in Fig. 4e is not caused by the increased content of Bi^0^, the Bi^0^ on the surface of Bi-H_*x*_/NF was oxidized into Bi^3+^ by treating the sample in the air at 80 °C for 0.5 h. The absence of Bi^0^ species on the surface of Bi-H_*x*_/NF after the treatment can be proved by XPS measurement (Supplementary Fig. 6). In the following, the controlled experiment for CO_2_ reduction was carried out on the sample after heat treatment. Compared with the activity of Bi-H_*x*_/NF in Fig. 4d, no performance degradation on CO production was found on Bi-H_*x*_/NF when the surface Bi^0^ species was replaced by Bi^3+^ (Fig. 4f), indicating that the improved activity of Bi-H_*x*_/NF in Fig. 4e cannot be attributed to the increased amount of surface Bi^0^.

Based on the above results, it can also speculate that the automatically formed Bi_2_O_3_ layer on Bi-H_*x*_/NF in the air plays negligible effect on the CO_2_ reduction activity. For the Bi-H_*x*_ covered by ~3 nm amorphous Bi_2_O_3_ layer, tunneling of the hot carrier to the out surface is still feasible due to the high energy of the hot charge carriers generated by LSPR and a high-density of defect states in the amorphous layer. A similar result has been observed on Bi catalysts for electrochemical H_2_ production and CO_2_ reduction in previous reports^22,24^. In addition, the specific surface areas of the two samples were studied by the Brunauer–Emmett–Teller (BET) method based on the nitrogen adsorption isotherm, showing that the BET surface areas of Bi-H_*x*_ and Bi are 3.3356 and 2.1039 m^2^ g^−1^, respectively. This result demonstrates that the surface area should not be the main factor for the enhanced CO_2_ reduction activity of Bi-H_*x*_/NF.

**Fig. 5:**
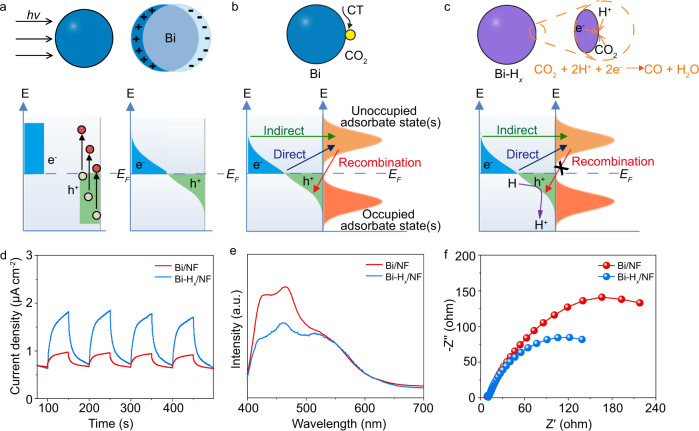
Effects of hydrogen storage on the photoinduced CT property of Bi. **a** Generation and relaxation of LSPR-induced carriers under light irradiation. The blue areas above the Fermi energy (*E*_F_) and the green areas below *E*_F_ represent the distributions of excited electrons and holes, respectively. **b** Schematic illustration of the transfer pathways of the LSPR-induced carriers in Bi through direct and indirect CT processes. **c** Graphical representation of the effect of the H stored in Bi-H_*x*_ on improving the carrier separation in the CO_2_ reduction reaction. **d** Photocurrent transient responses of Bi/NF and Bi-H_*x*_/NF catalysts under a 420-nm LED light irradiation at a bias of 1.07 V (vs. RHE). **e** The steady-state PL spectra of Bi/NF and Bi-H_*x*_/NF. **f** Nyquist plots of the Bi/NF and Bi-H_*x*_/NF catalysts at a bias of 1.07 V (vs. RHE).

**Fig. 6:**
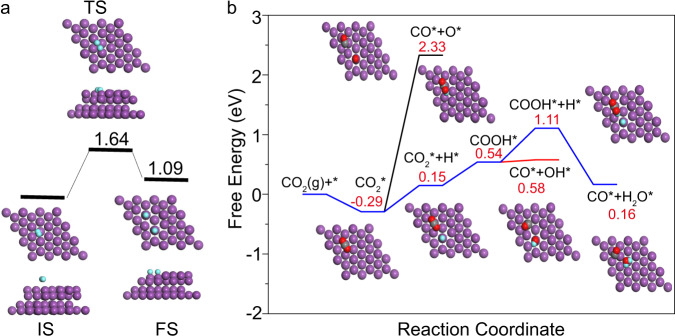
DFT calculations. **a** Reactant, transition state, and product of H_2_ → 2H* on Bi. **b** Free energy diagrams of CO_2_ → CO on Bi with (blue and red lines) and without (black line) hydrogen storage. The purple, gray, red, and blue balls represent Bi, C, O, and H atoms, respectively.

For the CO_2_ reduction process on Bi/NF with H_2_ gas, the photocatalytic interface must facilitate two sequence steps: (1) the adsorption and decomposition of the H_2_ to generate atomic H bound on the surface, and (2) the transfer of these adsorbed H atoms into electron–proton pairs to reduce CO_2_ (Fig. 4g). However, the two coupled steps (1 and 2) in both time and space preventing independent optimization of each, and the competitive adsorption of H_2_, may impede CO_2_ activation at the active sites^36^. Moreover, the decomposition of the H_2_ in step (1) will inevitably require certain energy input. By separating the above two steps (1 and 2), the present approach for CO_2_ conversion addresses the incompatibilities of functions (1) and (2) and bypasses the H_2_ decomposition process in the traditional CO_2_ reduction process with H_2_ (Fig. 4h). The improved CO_2_ reduction activity on Bi-H_*x*_/NF can be attributed to the atomic H atoms preformed on the surface, which not only improve photoinduced charge separation, but also lower the CO_2_ reduction barriers. These two positive effects will be closely discussed in the following Figs. 5 and  6, respectively.

To evaluate the effect of light on the catalytic CO evolution, the apparent quantum efficiency (AQE) as a function of the irradiation wavelength on Bi-H_*x*_/NF was examined, in which thermal contribution was considered in the calculation of AQEs (see details in “Methods” section). As shown in Fig. 4i, the AQEs at 420, 450, and 520 nm reach 0.066%, 0.048%, and 0.034%, respectively, confirming that the plasmonic excitation of Bi-H_*x*_/NF plays an important role in CO_2_ reduction. Obviously, the plasmonic photocatalyst of Bi-H_*x*_/NF is a type of catalyst that can combine light and thermal energy together for exciting CO_2_ reductions. To objectively understand the quantum efficiency of the plasmonic Bi catalysts, examples of thermal CO_2_ hydrogenation on metals, photocatalytic CO_2_ reduction based on plasmonic metals, and plasmonic metal coupled with semiconductor systems are compared. Although a temperature of 180 °C is needed in the present Bi-H_*x*_/NF system, this temperature is much lower than the thermochemical CO_2_ conversion process on metal catalysts (550–750 °C)^37^. Recently, plasmonic photocatalysis based on noble metal nanostructures was reported for CO_2_ reduction with a higher activity than the present Bi catalyst^30,31^. It should be indicated that the high activities on the noble metals were obtained under intense light illuminations (above 1 W cm^−2^). However, the present Bi catalyst can induce CO_2_-to-CO conversion with a relatively high CO evolution rate of 283.8 μmol g^−1^ h^−1^ under low-intensity LED light illumination (108.6 mW cm^−2^, around solar intensity). Moreover, in comparison to most semiconductor systems coupled with plasmonic metals, the present catalytic circle for CO_2_ reduction shows a higher photocatalytic activity, although some semiconductors with significantly high efficiencies were reported^1,4,29,38,39^. More details on the photocatalytic CO_2_ reduction efficiencies on semiconductor systems can be found in the recent review articles^1,4^. Rather than the catalytic efficiency, the main point of the present manuscript is to demonstrate a two-step reaction model for CO_2_ conversion by using a plasmonic Bi metal that entirely relies on nonprecious materials.

Recently, a growing number of plasmonic photocatalysts for driving chemical reactions by producing hot carriers under visible light illumination have been reported^40^. According to previous reports^41,42^, two possible mechanisms (indirect and direct) in chemical reactions driven by plasmonic metals are schematically shown in Fig. 5a, b. In the indirect mechanism, hot electron–hole pairs are generated in metal nanoparticles under light irradiation (Fig. 5b), forming a Fermi–Dirac-type distribution of hot electrons. In this distribution, hot electrons with sufficient energy can transfer to the unoccupied states of the adsorbed molecules, forming transient negative ions that then decay back to the metal. In the direct mechanism, electrons are assumed to be directly injected from the metal into the unoccupied state of the adsorbed reactant, leaving holes in the plasmonic metal (Fig. 5b). Compared with the indirect pathway, the formation of occupied states within the metal is avoided in the direct mechanism. Although some ramifications exist regarding the underlying mechanism in plasmonic photocatalysis, approaches that can improve the separation rates of hot electron–hole pairs should be beneficial for the charge injection process in both the indirect and direct mechanisms^41,42^.

Compared with the photogenerated charge in semiconductors, the lifetime of hot electrons produced by LSPR is much shorter^40^. Therefore, improving the separation efficiency of hot charges in plasma metals is particularly important. Similar to semiconductor photocatalysts, the electron transfer reactions on plasmonic Ag nanoparticles can be improved by using hole scavengers^41,42^. This phenomenon demonstrates that charge scavengers can also play an important role in driving photocatalytic reactions on plasmonic metals. Consequently, the role of the stored H atoms in the CO_2_ reduction of Bi-H_*x*_/NF is discussed based on the above consideration (Fig. 5c). Under light irradiation, hot electron–hole pairs are formed on irradiated Bi-H_*x*_ nanosheets through plasmonic excitation, as shown in Fig. 5a. The plasmonic hot holes are consumed by the H atoms stored in Bi-H_*x*_/NF (H + h^+^ → H^+^). In one operation, reactive H^+^ and e^−^ are synchronously generated on Bi-H_*x*_/NF and react with the activated CO_2_ molecules on the active sites (Fig. 5c). Based on this mechanism, suppression of the recombination of photoinduced carriers and enhancement of electron and proton donation are simultaneously realized by prepositioning H in the Bi catalyst, which accounts for the superior CO_2_ reduction performance on Bi-H_*x*_/NF through the proton-assisted electron transfer approach (CO_2_ + 2H^+^ + 2e^−^ → CO + H_2_O). Furthermore, the interdependent nature of H^+^ and e^−^ avoids the accumulation of H^+^ or e^−^ in the reaction process^15^, which is also beneficial for improving the performance of Bi-H_*x*_/NF. The fascinating point of Bi-H_*x*_/NF lies in the bifunctional H atoms. (1) They can act as hole scavengers to promote charge separation. (2) The produced protons are subsequently used as reactants that participate in the CO_2_ reduction reaction.

To provide evidence for the enhanced separation of photogenerated charge carriers on Bi-H_*x*_/NF, photoelectrochemical measurements were carried out. Figure 5d shows the transient photocurrent curves of the samples before and after hydrogen storage during four on-off cycles. As shown in Fig. 5d, the photocurrent intensity of Bi-H_*x*_/NF (0.95 μA cm^−2^) is approximately four times that of pristine Bi/NF (0.23 μA cm^−2^), indicating that a much lower recombination rate of photoinduced electrons and holes is indeed achieved by introducing hydrogen into Bi. To further study the separation of photoinduced electron–hole pairs under light illumination, steady-state photoluminescence (PL) analysis was performed on the two samples. As shown in Fig. 5e, the PL intensity of Bi-H_*x*_/NF is significantly decreased compared with that of Bi/NF, indicating that the incorporation of H atoms on Bi/NF has effectively suppressed the radiation recombination of charge carriers, which is helpful for CO_2_ reduction reaction on Bi-H_*x*_/NF. In addition, electrochemical impedance spectroscopy was used to investigate the CT resistance of the samples in the dark. The semicircular Nyquist plots are shown in Fig. 5f. Compared with Bi/NF, a much smaller semicircle diameter is detected for Bi-H_*x*_/NF, reflecting that the CT resistance is greatly reduced by storing hydrogen in Bi. The enhanced charge separation and transfer efficiencies play a role in facilitating photoinduced CO evolution on Bi-H_*x*_/NF.

In order to experimentally prove that the thermal-photocatalytic CO_2_ reduction on Bi-H_*x*_ was performed by the proton-assisted electron transfer approach, the following experiments were designed. First, CO_2_ reduction on Bi-H_*x*_/NF was performed at 180 °C without light illumination by using H_2_O (200 μL) as a proton source. The negligible CO production activity (Supplementary Fig. 7) demonstrates that photo-induced charges are the primary factor for CO_2_ reduction on Bi-H_*x*_/NF. Second, under the thermal-photocatalytic conditions, the formation of negligible CO on the bare Bi/NF without using proton sources (Supplementary Fig. 8) confirms that the CO_2_-to-CO conversion can hardly occur only with hot-electron transfer into unoccupied orbitals of adsorbed CO_2_. Based on the above two experiments, we can conclude that the thermal-photocatalytic CO production performance on Bi-H_*x*_/NF should originate from the synergetic effect between H^+^ and hot e^−^, which is accordant with previous reports^1–3^. The above experiments, taken together, provide solid evidence that the proton-assisted electron transfer approach indeed took place in the CO_2_ reduction process of Bi-H_*x*_/NF as shown in Fig. 5a–c, which is consistent with the density functional theory (DFT) calculations in Fig. 6.

To further understand the reaction mechanism, the effect of irradiation intensity on the thermal-photocatalytic CO_2_ reduction activity of Bi-H_*x*_/NF was examined (Supplementary Fig. 9). The strong dependence of the evolution rate of CO on the light intensity agrees well with the proposed mechanism that the synchronous production of reactive H^+^ and e^−^ pairs in Bi-H_*x*_ was achieved by the consumption of photogenerated holes by the stored H atoms. Moreover, the XPS curves of Bi-H_*x*_/NF after different thermo-photocatalytic reaction times were measured (Supplementary Fig. 10). It can be seen that the XPS band ascribed to the oxidation state of Bi progressively blue-shifts with increasing reaction time, which is consistent with the fact that the consumption of H species proceeds during the CO_2_ reduction reaction on Bi-H_*x*_/NF. As a result of the depletion of H species after 27 h reaction, the oxidation state of Bi in Bi-H_*x*_/NF returned to the state that is similar with Bi/NF without H storage (Supplementary Fig. 10). The reaction time-dependent XPS spectra also agree well with the proton-assisted electron transfer mechanism for CO_2_ reduction on Bi-H_*x*_/NF. In addition, based on the XPS analysis of Bi-H_*x*_/NF, no obvious change in the ratio of Bi^0^ and Bi^3+^ is observed after 27 h reaction, indicating the good stability of the catalyst under thermo-photocatalytic reaction conditions.

Experimental studies on CO_2_ reduction have shown that hydrogen storage in Bi plays an essential role in improving CO production compared with Bi in H_2_ gas (Fig. 4e). To further understand the difference between the performance of Bi-H_*x*_/NF and Bi/NF, DFT calculations were carried out. As discussed in the “Introduction” section, dissociation of H_2_ into hydrogen atoms is necessary for CO_2_ reduction. Therefore, the H_2_ dissociation pathway on Bi(001) was first calculated, and the potential energy curve is shown in Fig. 6a. The dissociation kinetic barrier (1.64 eV) for H_2_ on the pristine Bi(001) surface is high, which suggests that H_2_ dissociation is difficult in terms of kinetics. Furthermore, according to the calculation results, H_2_ dissociation is an endothermic process with an energy of 1.09 eV (Fig. 6a). Therefore, we can conclude that H_2_ decomposition is both kinetically and thermodynamically unfavorable on the Bi surface. Compared with the photoreduction of CO_2_ on Bi in H_2_ gas, the CO_2_ conversion on Bi-H_*x*_ avoids the unfavorable H_2_ dissociation step, and the stored H might directly react with CO_2_.

To further understand the role of the stored hydrogen in the CO_2_ reduction process, we calculated the free energy diagrams of CO_2_ reduction pathways on Bi/NF and Bi-H_*x*_/NF. The free energy curves of these pathways are depicted in Fig. 6b. As shown in Fig. 6b, direct decomposition of CO_2_ (CO_2_ → CO + O) with a high thermodynamic barrier of 2.33 eV occurs on the Bi surface. In contrast, if hydrogen-stored Bi (Bi-H_*x*_/NF) is used as the catalyst, then CO_2_ reduction will proceed via a proton-assisted electron transfer approach. In this process, one more reaction pathway of CO_2_* + H* → COOH* occurs. The thermodynamic energy barrier in this process is calculated to be 0.58 eV, which is lower than that of CO_2_ directly dissociating on the Bi surface, suggesting that atomic hydrogen plays a key role in the enhancement of the catalytic activity for CO_2_ reduction. Therefore, in addition to avoiding the H_2_ decomposition process, the storage of hydrogen would promote CO_2_ reduction on Bi-H_*x*_/NF by lowering the Gibbs free energy of the reaction pathway. According to the above DFT calculations, the hydrogen stored in Bi-H_*x*_/NF is further demonstrated to be responsible for the improved performance in the catalytic reduction of CO_2_, consistent with the experimental results shown in Fig. 4e.

As shown in the XPS spectrum of Bi-H_*x*_/NF in Fig. 2d, a part of Bi on the surface of Bi-H_*x*_/NF can be oxidized in the air. To clarify the effect of the oxidation state of Bi on CO_2_ reduction, DFT studies on oxidized Bi(001) were performed. As shown by the potential energy curves for H_2_ dissociation pathways on the oxidized Bi(001) surface (Supplementary Fig. 11a), the H_2_ molecule can be directly dissociated into atomic H* on oxidized Bi(001) with an energy gain of 1.30 eV and a large barrier of 1.86 eV, similar to that on the pristine Bi(001) surface. In addition, the free energy profiles associated with the reaction pathways of CO_2_ with and without stored hydrogen on the oxidized Bi(001) surface were calculated (Supplementary Fig. 11b). By comparison with the results in Fig. 6b, we can conclude that the energy profiles are not dramatically affected by the presence of Bi oxide. Consequently, the effect of oxidized Bi on the CO_2_ reduction activity of Bi-H_*x*_/NF and Bi/NF can be neglected.

## Discussion

Based on the above results, the entire catalytic reaction on Bi/NF is schematically shown in Fig. 1b, emphasizing that the reduction of CO_2_ in the present system occurs via two sequential steps: water splitting followed by CO_2_ reduction reactions. In the first step, electrochemical water splitting on Bi/NF leads to the simultaneous formation of O_2_ and Bi-H_*x*_/NF (as the reduced form of Bi/NF), similar to the light reaction in natural photosynthesis. In the second step, the reduction of CO_2_ to CO occurs on the reductive Bi-H_*x*_/NF by producing H^+^/e^−^ pairs under light irradiation, similar to the dark reaction in photosynthesis. After this reaction, the Bi-H_*x*_ recovers to Bi, which can be further used for storing and releasing H. In this strategy, the spatially separated water splitting and CO_2_ reduction reactions are integrated by using Bi as an electron–proton-transfer mediator. As a result, catalytic overall splitting of CO_2_ on Bi/NF is realized by the two sequential reactions, which correspond to the light and dark reactions in natural photosynthesis (Fig. 1a). Therefore, the whole CO_2_ conversion on Bi/NF with the help of H_2_O can be regarded as artificial photosynthesis. In fact, the water-splitting process can be powered by renewable electricity (e.g., solar and wind). Consequently, the catalytic cycle on Bi/NF for overall CO_2_ splitting can be driven by renewable energy.

It should be indicated that the low FE of the Bi catalyst (20.9%) in the decomposition of H_2_O is a drawback for it. However, the present manuscript does not aim to develop high-efficiency electrocatalysts, but rather demonstrates a two-step reaction model mediated by plasmonic Bi-H_*x*_ for CO_2_ conversion by mimicking photosynthesis. Improving the electrochemical performance of Bi is important for reducing the overall energy input in the present two-step CO_2_ reduction process, which is the subject of ongoing investigations in our laboratory.

Although electrocatalytic reduction of CO_2_ into formate (HCOO^−^) on Bi electrode can reach a much higher FE efficiency than the electrochemical H_2_O splitting on the present Bi/NF, higher overpotentials between −0. 67 and −0.87 V (vs. RHE) were used in previous reports^43–45^. Furthermore, the direct reduction of CO_2_ by the electrochemical approach is not a perfect technology because it still suffers from low product concentrations in the mixture of traditional liquid electrolytes^46^. As a result, the product cannot be directly used without further purification. However, the purification of the low concentration liquid fuel in electrolytes not only compromises energy efficiency, but also is a technical challenge^46^.

It is well known that different from acidic form, formate has no obvious usage^47^. In contrast, CO gas is a critical feedstock for directly synthesizing a variety of chemicals in industry^47^. Therefore, compared with electrochemical CO_2_ reduction on Bi, the CO_2_-to-CO conversion achieved on the present Bi catalyst has great potential in practical applications. Furthermore, the electrocatalytic approach displays poor solubility for gaseous substrates and limits the rate of the substrate bond activation. Therefore, it is difficult to perform direct CO_2_ reduction in the electrocatalytic approach^36^. In addition, although the gas product and O_2_ generation in electrocatalytic CO_2_ conversion could also be spatially separated by a membrane, the used membrane is expensive and tends to be degraded at low current densities^17^. However, the above three drawbacks do not exist in the present catalytic system for CO_2_ reduction. Based on our understanding, there is a noticeable absence of a consummate material that can solve all of these challenges in CO_2_ reduction together.

In addition to using H_2_ and H_2_O as proton sources, developing hydrides as hydrogen storage materials are believed to be an emerging field for the reduction of CO_2_. As attractive candidates, hydrides of silicon (Si) nanostructures have been sufficiently studied for converting CO_2_ to useful chemicals, such as formaldehyde, CO, and methanol^48–51^. In these processes, the Si hydrides were used as reducing agents to react stoichiometrically with CO_2_. However, achieving the catalytic conversion of CO_2_ on Si hydrides is difficult because the formation of inactive Si–OH and Si–O–Si groups after CO_2_ reduction prevents recovery of Si surface hydrides^52^. Although catalytic CO_2_ conversion on silicon–hydride nanosheets was realized, the use of H_2_ gas as a reductive agent and loading of precious palladium nanoparticles are simultaneously needed^52^. Our CO_2_ conversion strategy is important because overall CO_2_ splitting with water is achieved by using a low-cost, environmentally friendly Bi catalyst as a hydrogen shuttle to bridge water splitting and CO_2_ reduction reactions, which provides an effective strategy for designing CO_2_ reduction catalysts by separately mimicking the light and dark reactions in natural photosynthesis. Essentially, the two-step strategy successfully enables reactive hydrogen adatoms to concentrate on the active site, which provides a high driving force for CO_2_ reduction on Bi-H_*x*_/NF. More importantly, in the artificial photosynthesis, the O_2_ separation procedures encountered in the traditional overall CO_2_ splitting systems are successfully avoided.

## Methods

The Bi-H_*x*_ sample was loaded on NF by electrochemical method. Prior to Bi-H_*x*_ loading, NF (thickness: 1.0 mm, area: 1 × 4.5 cm^2^) was cleaned in acetone, hydrochloric acid (0.1 mol L^−1^), and deionized water under ultrasonication for 20 min each. Then, it was dried by flowing air. Electrodeposition of Bi nanosheets on the NF was carried out in a standard three-electrode system containing an aqueous solution of 0.013 M Bi(NO_3_)_3_ and 1 M HNO_3_. The NF, platinum wire, and a Ag/AgCl electrode were used as the working electrode, counter electrode, and reference electrode, respectively. The synthesis of Bi/NF was performed at a reduction potential of 0.16 V (vs. RHE) for 15 min. To synthesize Bi-H_*x*_/NF, Bi/NF was treated in the above system using KOH (1 M) as the electrolyte for 2–12 min at a voltage of −0.18 V (vs. RHE). In this process, the generated O_2_ gas was quantified by Neofox-GT oxygen probe (Ocean opticals). Finally, the obtained Bi-H_*x*_/NF electrodes were immediately washed with deionized water and then dried at 80 °C for 2 h in Ar gas.

The content of Bi loaded on NF was analyzed by ICP. XRD patterns were recorded on a D8 Advance Bruker diffractometer with Cu Kα radiation. Data were collected from 20° to 80° in 2*θ* with a scan rate of 0.02° steps s^−1^. The morphology of the prepared samples was characterized by SEM (JSM-6510 microscope). TEM and HRTEM images were acquired on a TEM (JEM 2100F). XPS data were measured on a Kratos AXIS Supra spectrometer using monochromatic AlKα radiation as the excitation source. All binding energies were referenced to the C1*s* peak (284.8 eV) arising from adventitious carbon. UV–visible absorption spectra were recorded on a Shimadu UV-2600 spectrometer. PL spectra were obtained on a fluorescence spectrophotometer (F-7000, Hitachi, Japan) at room temperature. The BET surface area was determined by nitrogen adsorption on a Micromeritics ASAP 2460 nitrogen adsorption apparatus. An HP 5973 GC-mass spectrometer was employed to analyze the ^13^CO generated from the ^13^CO_2_ isotopic experiment. Electrochemical measurements were performed using an electrochemical analyzer (CHI660E, Chenhua, Shanghai) with a standard three-electrode system in the normal atmosphere. The prepared sample, Pt wire, and a saturated Ag/AgCl electrode were used as the working electrode, counter electrode, and reference electrode, respectively. CV curves and Nyquist plots were obtained at a scan rate of 50 mV s^−1^ in 1 M KOH aqueous solution. The observed potentials were converted to RHE based on:

6 $$E_{{\mathrm{RHE}}} = E_{{\mathrm{Ag}}/{\mathrm{AgCl}}} + 0.197 + 0.059 \times {\mathrm{pH}}.$$

FE of O_2_ was calculated through:

7 $${\mathrm{FE}} = znF/Q,$$

where *z* is the number of electron transfer, *n* is the total moles of O_2_, *F* is the Faraday constant of 96485 C mol^−1^, and *Q* is the total amount of electricity.

CO_2_ reduction reactions of the synthesized samples were carried out in a 300 mL closed gas system with a quartz window on top. A 420-nm LED light was employed as the light source. The temperature was measured by a thermocouple and controlled by a heating source. The Bi-H_*x*_/NF sample was cut into a rectangular shape of 1 × 2 cm^2^ for activity tests. The CO_2_ reduction reactions were carried out in a gas mixture of CO_2_ and Ar. The mole ratio of CO_2_:Ar was 1:5. For detecting CO and CH_4_, the gas products on Bi-H_*x*_/NF were analyzed by a GC (Agilent 7890B) with a thermal conductivity detector. The GC was equipped with a 5 Å molecular sieve column using He as the carrier gas. For detecting CH_3_OH, the gaseous products from the reactor were analyzed by an offline GC (Fuli Corp., China) equipped with a flame-ionized detector. The equipped column was TDX-01. For comparison, CO_2_ reduction reactions on Bi/NF were also performed in a gas mixture of CO_2_ (50 mL), H_2_ (0.5 mL), and Ar (249.5 mL), with the other conditions unchanged.

The AQEs for CO evolution on Bi-H_*x*_/NF were measured under the same catalytic reaction conditions with irradiation of different wavelengths (420, 450, 520, and 590 nm). The light intensity (*W*_l_) was monitored by a PL-MW200 photoradiometer (Perfect Light, Beijing, China). Thermal contribution at 180 °C was considered in the calculation of AQEs. The radiative power of heat per unit area can be described by the Stefan–Boltzmann law^53^,

8 $$Q = \sigma \left( {T_{\mathrm{r}}^4 - T_0^4} \right),$$

where *T*_r_ is the reaction temperature in K and *σ* is Stefan–Boltzmann constant (5.67 × 10^−8^ W m^−2^ K^−4^). The maximal work done by the thermal energy was calculated by using the Carnot equation^54^,

9 $$W_{\mathrm{h}} = Q\left( {1 - T_0/T_{\mathrm{r}}} \right),$$

where *T*_0_ is the ambient temperature for performing the CO_2_ reduction (299.15 K). Based on the above two equations, the real contribution of heat at 180 °C in CO_2_ reduction was calculated to be 65.8 mW cm^−2^. The AQE was calculated by:

10 $${\mathrm{AQE}}\;\left( \% \right) 	= \frac{{{\mathrm{Number}}\;{\mathrm{of}}\;{\mathrm{reacted}}\;{\mathrm{electrons}}}}{{{\mathrm{Number}}\;{\mathrm{of}}\;{\mathrm{incident}}\;{\mathrm{photons}}}} \times 100\% \\ 	= \frac{{{\mathrm{Number}}\;{\mathrm{of}}\;{\mathrm{evolved}}\; {\mathrm{CO}}\; {\mathrm{molecules}} {\,}\times 2}}{{{\mathrm{Number}}\;{\mathrm{of}}\;{\mathrm{incident}}\;{\mathrm{photons}}}} \times 100\% \\ 	= \frac{{M \times N_{\mathrm{A}} \times 2}}{{\frac{{W \times A \times t \times \lambda }}{{h \times c}}}} \times 100\% \\ 	= \frac{{M \times N_{\mathrm{A}} \times 2}}{{\frac{{\left( {W_l + W_h} \right) \times A \times t \times \lambda }}{{h \times c}}}} \times 100\%,$$

where *M* is the CO evolution rate (mol s^−1^), *N*_A_ is Avogadro constant, *W* is the total energy input (W), *A* is irradiation area (m^2^), *t* is the time of light illumination (s), *λ* is the corresponding wavelength (m), *h* is 6.62 × 10^−34^ J s^−1^, and *c* is 3.0 × 10^8^ m s^−1^.

The electronic structure and free energy calculations were performed using spin-polarized DFT based on the Vienna ab initio simulation package^55^. PAW potentials were selected to describe ion core and valence electron interactions^56^. For the exchange-correlation functional, the generalized gradient approximation with the Perdew–Burke–Ernzerhof functional was adopted^57^. A kinetic energy cut-off of 400 eV was used with a plane-wave basis set. The integration of the Brillouin zone was conducted using a 1 × 1 × 1 Monkhorst–Pack grid^58^. To explain the reaction activity from a kinetic aspect, the transition state structures and the reaction pathways were located using the climbing image nudged elastic band method^59^. The minimum energy path was optimized using a force-based conjugate-gradient method until the energy converged to 1.0 × 10^−4^ eV atom^−1^ and the maximum force was <0.05 eV Å^−1^. Spin polarization and van der Waals (vDW) forces were considered in our current study using the vDW-DF method. The Bi(001) surface was obtained by cutting bulk Bi along the [001] direction. A 3 × 3 supercell with four layers was chosen as our model. During the geometry optimization, the atoms in the top three layers were allowed to relax, while the bottom layer was fixed. A vacuum layer of 15 Å was used along the *c*-direction normal to the surface to avoid periodic interactions.

To illustrate the CO_2_ reduction reaction activity from a thermodynamic aspect, the free energy diagrams were estimated using:

11 $${\Delta}G = {\Delta}E + {\Delta}ZPE - T{\Delta}S,$$

where Δ*E* is the total energy change based on the DFT calculations, ZPE and *S* are the zero-point energy and entropy, respectively, and *T* is the temperature (here, 298.15 K is selected). The free energy of (H^+^ + e^−^) at standard conditions was assumed to be the energy of 1/2 H_2_^60^.

## Supplementary information

Supplementary Information

Peer Review File

## Data Availability

The data that support the findings of this study are available from the corresponding author upon reasonable request.
