# Supplementary material for: Boosting thermo-photocatalytic CO2 conversion activity by using photosynthesis-inspired electron-proton-transfer mediators
Source: Nat Commun. 2021 Jan 5;12:123. doi: 10.1038/s41467-020-20444-1 (PMC7785748; doi:10.1038/s41467-020-20444-1)
Supplement: Supplementary file 1 — Supplementary Information [file 41467_2020_20444_MOESM1_ESM.pdf]

Supplementary information for

Boosting thermo-photocatalytic CO_2 conversion activity by using photosynthesis-inspired electron-proton-transfer mediators

Yingxuan Li[1], Danping Hui[1], Yuqing Sun[1], Ying Wang[2], Zhijian Wu[2], Chuanyi Wang[1] & Jincai Zhao[3]

[1]School of Environmental Science and Engineering, Shaanxi University of Science and Technology, Xi'an 710021, China

[2]State Key Laboratory of Rare Earth Resource Utilization, Changchun Institute of Applied Chemistry, Chinese Academy of Sciences, Changchun 130022, China

[3]Key Laboratory of Photochemistry, Beijing National Laboratory for Molecular Sciences, Institute of Chemistry, Chinese Academy of Sciences, Beijing, 100190, China

*****To whom correspondence should be addressed. E-mail: Y.L. (email: liyingxuan@sust.edu.cn); Y.W. (email: ywang_2012@ciac.ac.cn).

Supplementary information

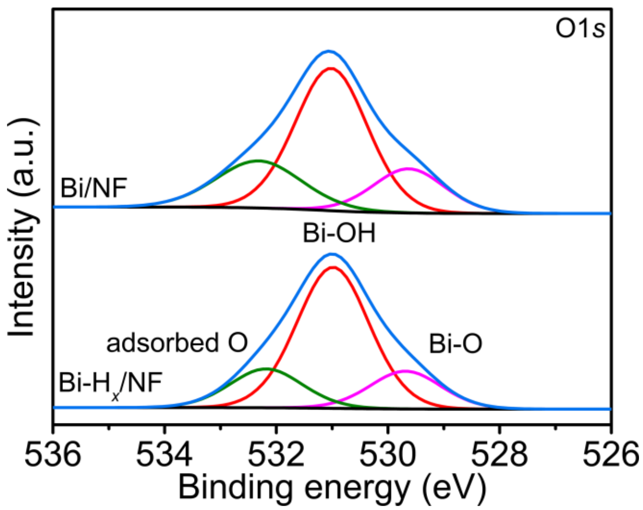

Supplementary Fig. 1 Properties of surface O on Bi/NF and Bi-H$_x$/NF. High-resolution O 1*s* XPS spectra of Bi/NF and Bi-H$_x$/NF.

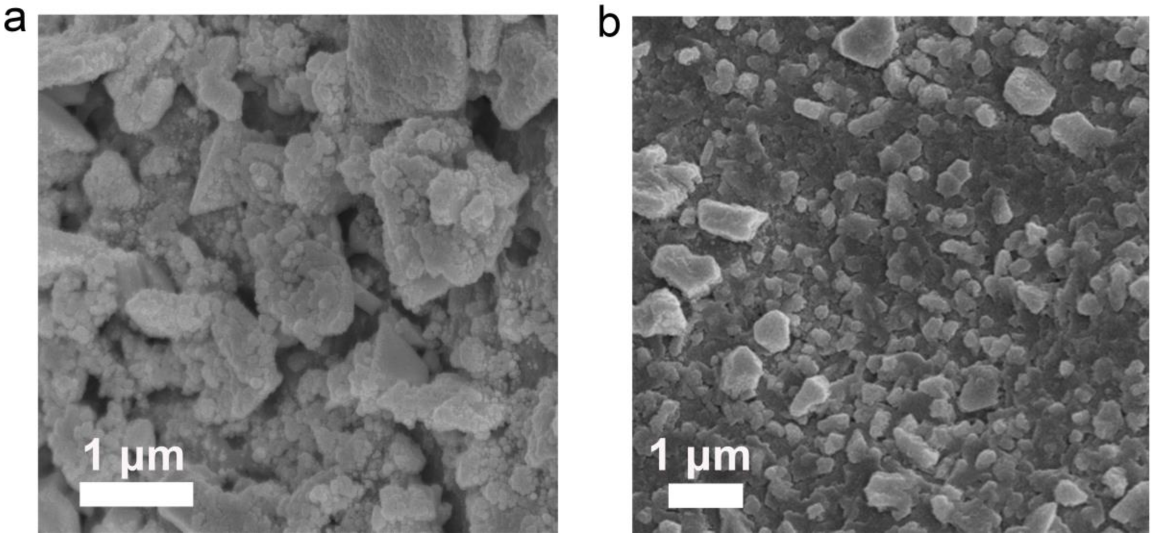

Supplementary Fig. 2 Morphologies of the samples. a, b SEM images of Bi/NF at the deposition time of 1 and 4 min.

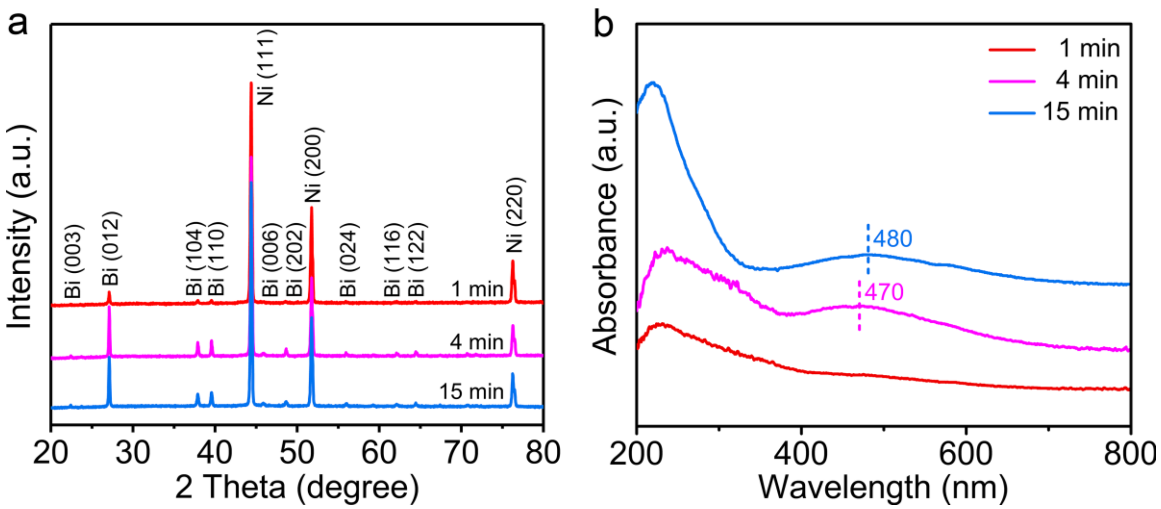

Supplementary Fig. 3 Structure and optical properties of the samples. a, b XRD patterns and UV-visible adsorption spectra of Bi/NF samples prepared at different deposition time.

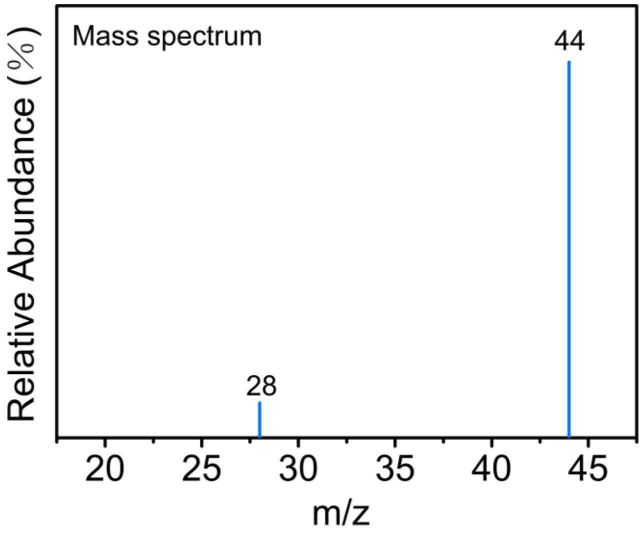

Supplementary Fig. 4 Isotopic labelling test. Mass spectrum from GC-MS analysis of the CO generated in the catalytic CO_2 reduction reaction using $^{12}CO_2$.

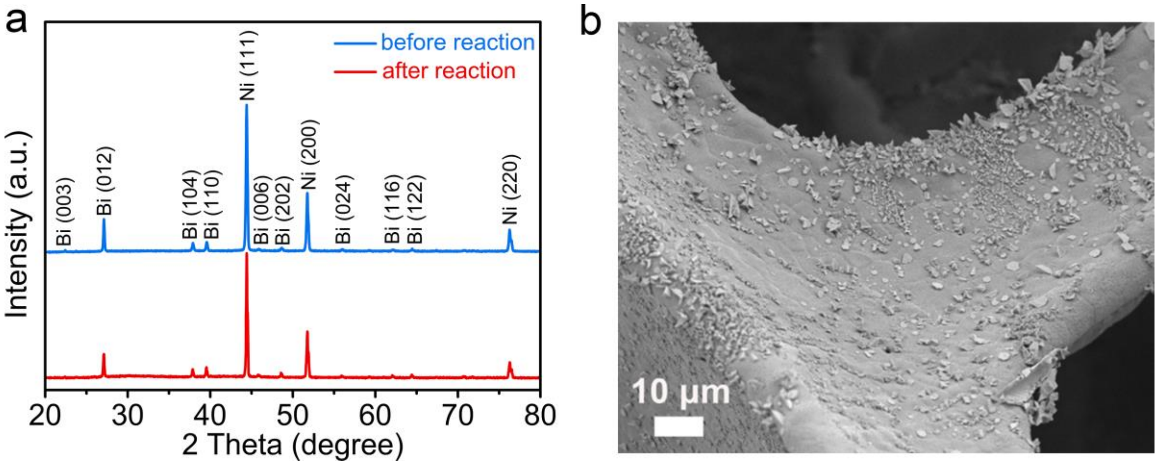

Supplementary Fig. 5 Characterization of Bi-H$_x$/NF after CO_2 reduction reaction. a, b XRD pattern and SEM image of Bi-H$_x$/NF after 81 h catalytic reaction.

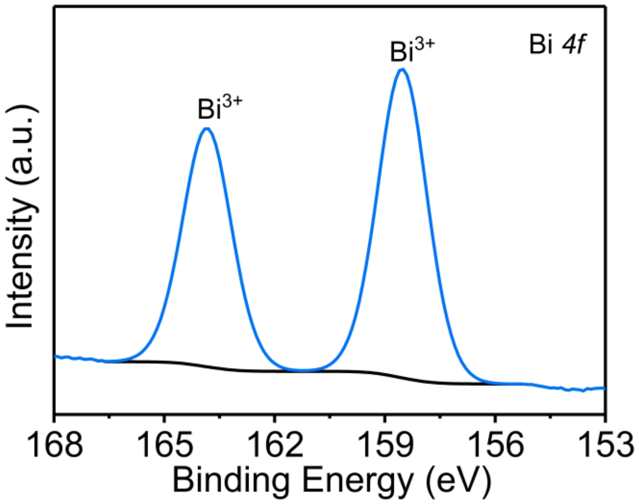

Supplementary Fig. 6 Property of surface Bi on Bi-H$_x$/NF. High-resolution XPS spectra of Bi 4*f* of the Bi-H$_x$/NF sample treated in air at 80 °C for 0.5 h.

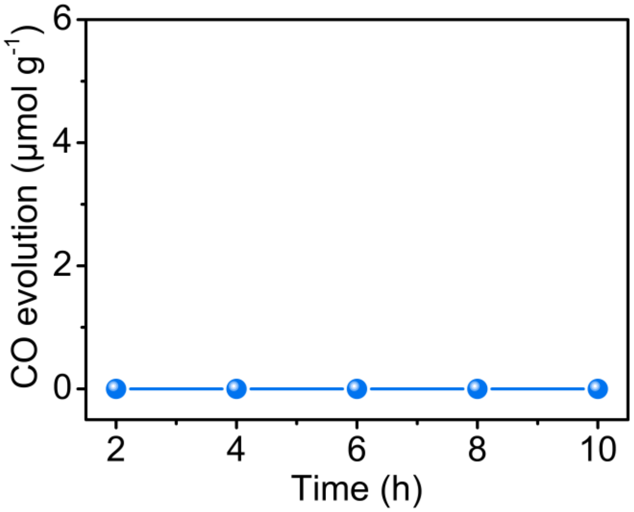

Supplementary Fig. 7 Catalytic activity of Bi-H_x/NF for CO_2 reduction with only heat input. CO production on Bi-H_x/NF at 180 °C without light illumination by using H_2O (200 μL) as proton source.

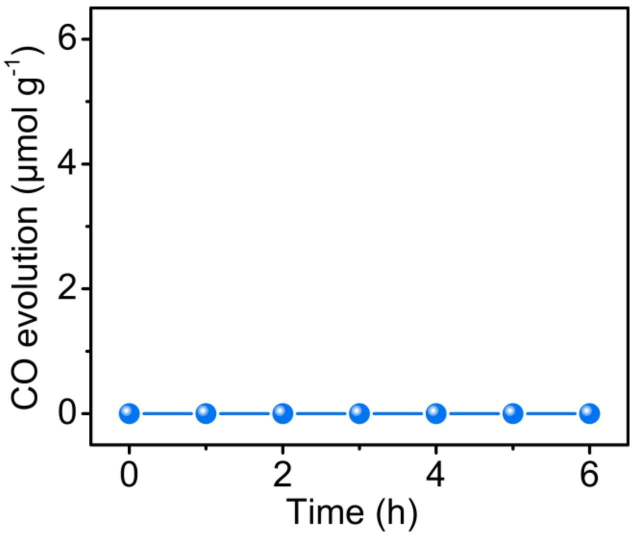

Supplementary Fig. 8 Thermal-photocatalytic activity of Bi/NF for CO_2 reduction. CO production on Bi/NF without using proton sources under the thermal-photocatalytic conditions.

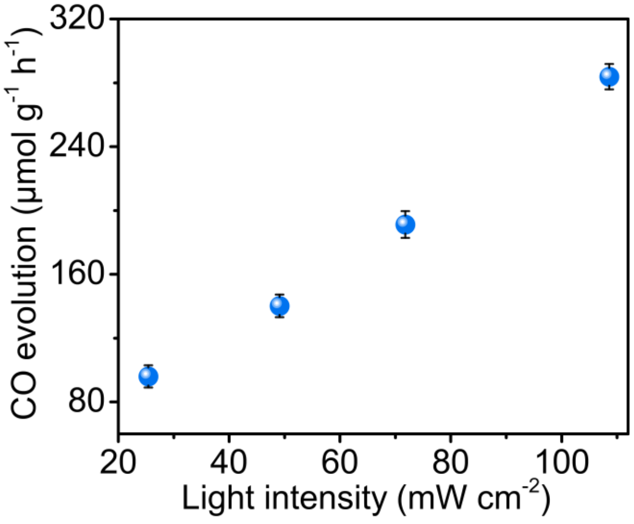

Supplementary Fig. 9 Light intensity dependent catalytic activity of Bi-H$_x$/NF. Effect of light intensity on CO evolution rate over Bi-H$_x$/NF at 180 °C ($n = 3$, error bars: standard deviation).

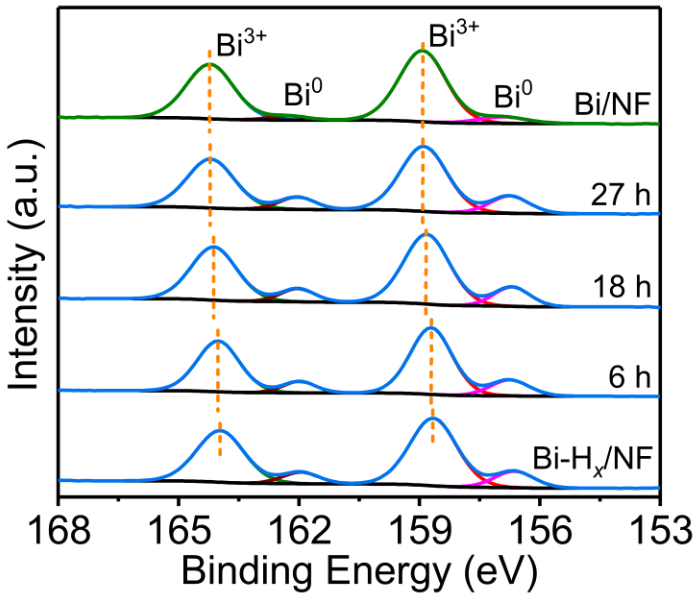

Supplementary Fig. 10 Properties of surface Bi on Bi-H$_x$/NF. XPS spectra of Bi-H$_x$/NF after different thermo-photocatalytic reaction time.

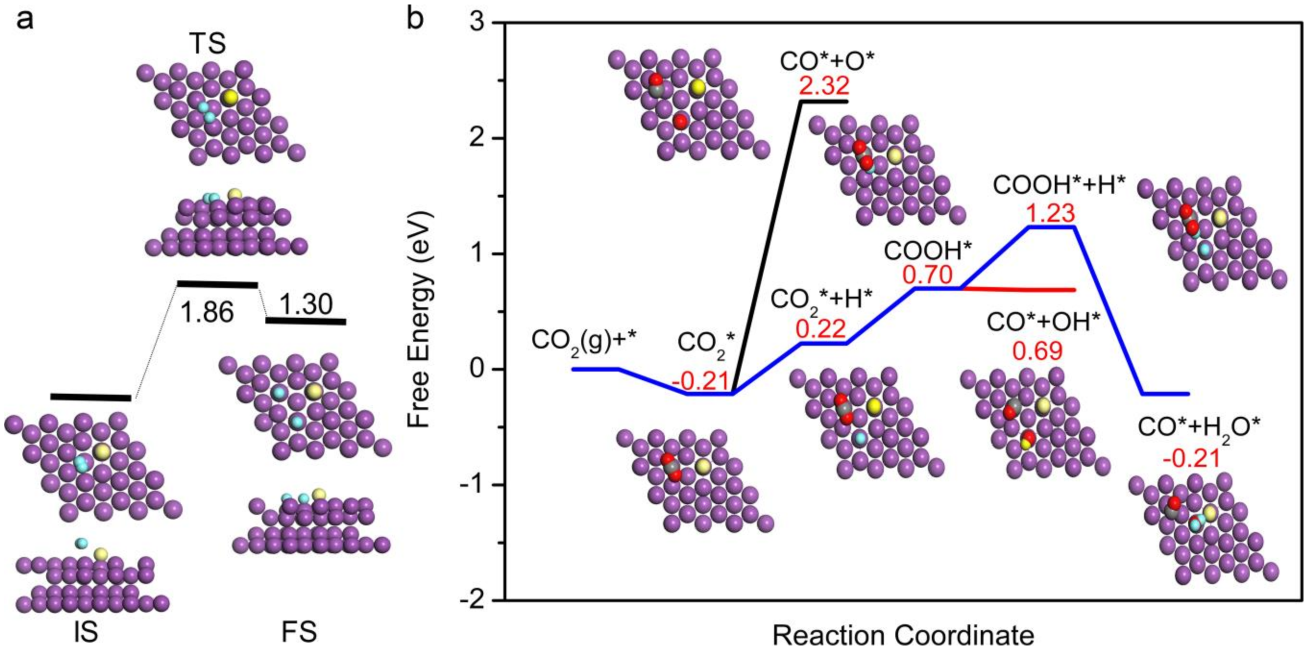

Supplementary Fig. 11 DFT calculations. a Reactant, transition state, and product of H_2 → 2H* on oxidized Bi (Bi-O). **b** Free energy diagrams of CO_2 → CO on oxidized Bi (Bi-O). The purple, gray, red and blue balls stand for Bi, C, O and H atoms, respectively, and the oxygen in oxidized Bi is highlighted in yellow.
